# Clinical evaluation of different bi-layer biomimetic strategies of composite resin in large class I cavities: an 18-month randomized clinical trial

**DOI:** 10.1186/s12903-026-08403-6

**Published:** 2026-05-01

**Authors:** Mohammed Abd El Ghany Mohammed, Mohammed Abdallah Hassan, Kholood El Sayed Morsy

**Affiliations:** 1 Restorative Department, Faculty of Dentistry, Tanta University, Tanta, Egypt; 2 Faculty of Oral and Dental Medicine, Alsalam University, Tanta, 6618002 Egypt

**Keywords:** Fiber-reinforced composite, Resin-modified glass ionomer, Flowable composite, Randomized trial, Posterior restoration, Split-mouth design, Biomimetic, Class I

## Abstract

**Objective:**

The aim of this study was to conduct a randomized, double-blind, split-mouth comparative clinical trial to clinically assess and compare different bi-layer biomimetic strategies of composite resin in large class I cavities over an 18-month period.

**Materials & methods:**

A total of 160 posterior composite restorations in 40 participants of age 35–45 years old were enrolled in the study based on inclusion criteria. Each patient received four different bilayer biomimetic bases in four equal groups (*n* = 40) as follows: Group I: short-fiber reinforced composite (everX Posterior, GC Corporation, Tokyo, Japan); Group II: long-fiber reinforced composite (polyethylene fibers, Ribbond Inc., USA); Group III: Resin-Modified Glass Ionomer Cement (RMGIC) (Riva SDI, Bayswater, VIC, Australia); and Group IV: flowable composite (G-aenial universal Flo, GC Corp., Tokyo, Japan). A 2 mm surface layer of nanohybrid composite resin (Tetric Evo Ceram, Ivoclar Vivadent) was applied to cover the biomimetic base in all groups. Occlusal Class I cavities were prepared according to caries extension with a cavity depth of 4–5 mm. No bevels were prepared. Clinical assessment was carried out according to the World Dental Federation (FDI) by two calibrated evaluators at baseline and at 6, 12, and 18 months for aesthetic properties (surface and marginal staining), functional properties (fracture and retention, marginal adaptation), and biological properties (postoperative sensitivity, secondary caries). Statistical analysis employed Friedman and Kruskal-Wallis tests. The level of significance was established at α = 0.05 across all tests.

**Results:**

Forty patients with a total of 160 restorations were evaluated in line with FDI at the end of the study with 100% recall rates. No fracture or secondary caries was reported over an 18-month evaluation period. The intragroup (Friedman test) and intergroup (Kruskal-Wallis test) comparisons revealed no statistically significant differences among short fiber reinforced composite, long fiber reinforced composite, resin-modified glass ionomer cement, and flowable composite for the assessed criteria over time (*p* > 0.05).

**Conclusion:**

All tested bilayer biomimetic composite resins have comparable excellent short-term clinical performance by the end of the study period.

**Trial registration:**

The protocol of the current study was approved by the Research Ethics Committee of the Faculty of Dentistry, Tanta University, Egypt, with approval number #R-RD-03-25-3194. This study was registered at www.clinicaltrials.gov with the identification number NCT07285772 on 15/12/2025—‘retrospectively registered.’

## Introduction

Over the past few decades, growing demands have significantly increased the use of direct, light-activated resin composites in both anterior and posterior teeth. They are considered as an essential treatment option in restorative dentistry due to the increasing demand for high quality esthetic results in daily practice. This has been motivated by factors such as patient demand, an increased desire for minimally invasive restorations and more reliable dental adhesive systems [1].

The durability of resin composite restorations is still threatened by serious challenges like polymerization shrinkage and marginal microleakage, even with the ongoing advancement of resin composite materials. The shrinkage of the resin that occurs during the light curing process creates internal and interfacial stresses resulting in the formation of a gap and subsequent marginal leakage. This permits fluid, bacteria, and ions to enter via the tooth restoration interface, leading to pulpal irritation, recurrent decay, post-operative pain and restorative failure [2].

The various restorative techniques and composite resin material properties have demonstrated a significant effect on the adhesion of restorations to tooth structures particularly when dealing with cavities with high C-factor as Class I cavities which generates the highest polymerization shrinkage stresses. However, the polymerization shrinkage and associated adverse clinical implications can be minimized using different biomimetic bi-layer strategies [3].

Based on the concepts of modern restorative dentistry, the restoration and the tooth should be joined together as a mono block, with both structures joining mechanically and adhesively to withstand the repeated forces of mastication for a long time. The biomimetic approach is very important in restorative dentistry. The technique involves biomimetic bi-layer composite restorations that mimic the naturally occurring bi-layered tooth structures (enamel and dentin) by substituting missing tooth structures with restorative materials that have biomechanical properties comparable to those of the sound teeth structures [4].

The application of an intermediate layer with modulus of elasticity similar to dentin beneath the restorative resin restoration has been shown to relieve polymerization contraction stresses by 20%–50% according to the “elastic cavity wall concept [5]. Numerous studies have assessed the use of fiber reinforced composite, glass ionomers, or flowable composites as a dentin mimicking materials and stress-absorbing layer. Flowable composites are materials that have a lower viscosity, which allows them to flow readily, adapt well to the surface of the tooth, and also function as a flexible inter-mediate layer that absorbs stresses throughout the process of polymerization shrinkage [6–8].

The use of glass ionomer cement as dentin substitute in closed sandwich technique has many benefits include coefficient of thermal expansion similar to that of dental structures, bacteriostatic function due to its fluoride-releasing ability, provides chemical adhesion to the tooth, micromechanical bond to the overlying resin, it has a lower modulus of elasticity and can act as an elastic buffer or a stress-breaking barrier and reduces polymerization shrinkage [9].

As a potential method for enhancing the fracture toughness of cavities that are subjected to high levels of stresses, the utilization of fiber reinforcement in resin composite restorations has been proposed. Numerous prior investigations have demonstrated the efficacy of fiber-reinforced composite restorations (FRCs). By adjusting their orientation, fibers can control the stresses that result from polymerization shrinkage. So, compared to conventional resin composites, there is less marginal microleakage as suggested by in vitro evidence; however, clinical confirmation remains limited. These composites could be better suited for extensive direct restorations since the fiber structure improves their mechanical properties [10].

A short-fiber reinforced resin composite (SFRC) called EverX Posterior, manufactured by GC Corporation in Japan, can be used as a dentine substitute. Its fibers resemble dentine’s collagen fibers and are randomly oriented discontinuous short electric (E)-glass fibers. The volumetric shrinkage of this composite material is much lower than that of competing materials. The direction of the fibers’ long axis reduces the polymerization contraction of SFRC. Also, the fracture load of a restoration is increased when a bi-layered structure is used, which consists of a fiber-reinforced composite substructure and a traditional restorative composite surface layer [11].

Long continuous ultra-high-molecular-weight polyethylene fibers with a cross-linked leno weave pattern and locked nodal crossings are known as Ribbond Ultra fibers, and they are manufactured by Ribbond Inc., Seattle, Washington, USA. Also, the stress values are reduced since the energy from the forces can be absorbed and distributed favorably due to the fibers’ orientation in multiple directions. In addition, placing the Ribbond beneath the composite restoration has been demonstrated to promote marginal adaption and fracture resistance by shielding the adhesive interface from the stresses caused by polymerization in large and deep cavities [12].

Clinical testing is essential for determining the durability of the restorative materials, as it surpasses in vitro screening. There is an enormous variance between in vivo and in vitro circumstances, even in experiments that use meticulously simulated clinical scenarios. It is not possible to replicate the complex interplay of oral environment, tooth substrate, and other clinical variables in a controlled laboratory setting [13].

There is currently a lack of direct clinical evidence comparing these four tested materials that comprised this present study in Class I restorations, especially under normal occlusal loads in vital teeth. No existing study evaluates their performance over 18 months using standardized clinical criteria, leaving uncertainty regarding which material offers the most predictable outcomes. Many studies using fiber-reinforced composites focus on endodontically treated teeth, not routine vital Class I cavities. This limits generalizability to common daily clinical situations. The null hypothesis of this study is that there is no significant difference in the clinical performance in terms of restoration retention/fracture, marginal adaptation, staining (primary outcome), postoperative hypersensitivity, and secondary caries (secondary outcome) between different bi-layer biomimetic strategies of composite resin in large class I cavities over 18 months.

## Materials and methods

The chemical composition, manufacturers, and websites of the materials utilized in this study are listed in Table 1.

**Table 1:** Materials used in the study

Materials	Chemical compositions	Manufactures	Batch number	Website
EverX Posterior (Short Glass Fiber Reinforced Composite) (SFRC)	resin matrix: bisphenol A-glycidyl methacrylate (Bis-GMA), triethylene glycol dimethacrylate (TEGDMA), Polymethyl Methacrylate (PMMA). Filler Content: Silanated E-glass fiber Silicon dioxide, Barium glass, filler loading:76 Wt%,57 Vol%	GC Corporation, Tokyo, Japan	2,504,151	https://www.gc.dental/europe/en/products/everxposterior
Ribbond-Ultra (Ultra-High Molecular Weight Long Continuous Polyethylene Fibers)	Silanized Polyethylene Fibers	Ribbond Inc., Seattle, WA 98,101, USA	*+D758T0/$$7272/16D20251126/U*	https://ribbond.com/
Riva SDI (Resin-Modified Glass Ionomer Cement)	2-Hydroxyethyl methacrylate (20–25%) Dimethacrylate cross-linker (10–25%) Acidic monomer (10–20%) Fluoroaluminosilicate glass powder (95–100%)	SDI, Bayswater, VIC, Australia	1,210,951 A	https://www.sdi.com.au/au/product/riva-light-cure/
G-aenial universal Flo (Flowable composite)	Triethylene glycol dimethacrylate (TEGDMA), Urethane Dimethacrylate (UDMA), Bisphenol A Ethoxylated Dimethacrylate (Bis-MEPP), SO_2_ (16 nm), Strontium glass (200 nm) Filler loading:69 wt %, 50% vol	GC Corporation, Tokyo, Japan	220,203 A	https://www.gc.dental/america/products/operatory/composite-restoratives/g-aenial-universal-flo
Tetric Evo Ceram (nanohybrid composite resin)	Resin matrix: Bisphenol-A Ethoxylated Dimethacrylate (Bis-EMA), bisphenol A-glycidyl methacrylate (Bis-GMA) and triethylene glycol dimethacrylate (TEGDMA) Pre-polymerized fillers and silica Filler loading: 75% vol	Ivoclar Vivadent, Schaan, Liechtenstein	Z011D1	https://www.ivoclarvivadent.com
Tetric N-Bond (Universal One step adhesive)	Silane, water, initiators, ethanol, Vitrebond copolymer, 2-hydroxyethyl methacrylate (HEMA), bisphenol A diglycidyl ether dimethacrylate (Bis-GMA), decamethylene dimethacrylate, 10-methacryloyloxydecyl dihydrogen phosphate (MDP)	Ivoclar Vivadent, Schaan, Liechtenstein	Z056K7	https://www.ivoclarvivadent.com

### Study design

**Fig. 1:**
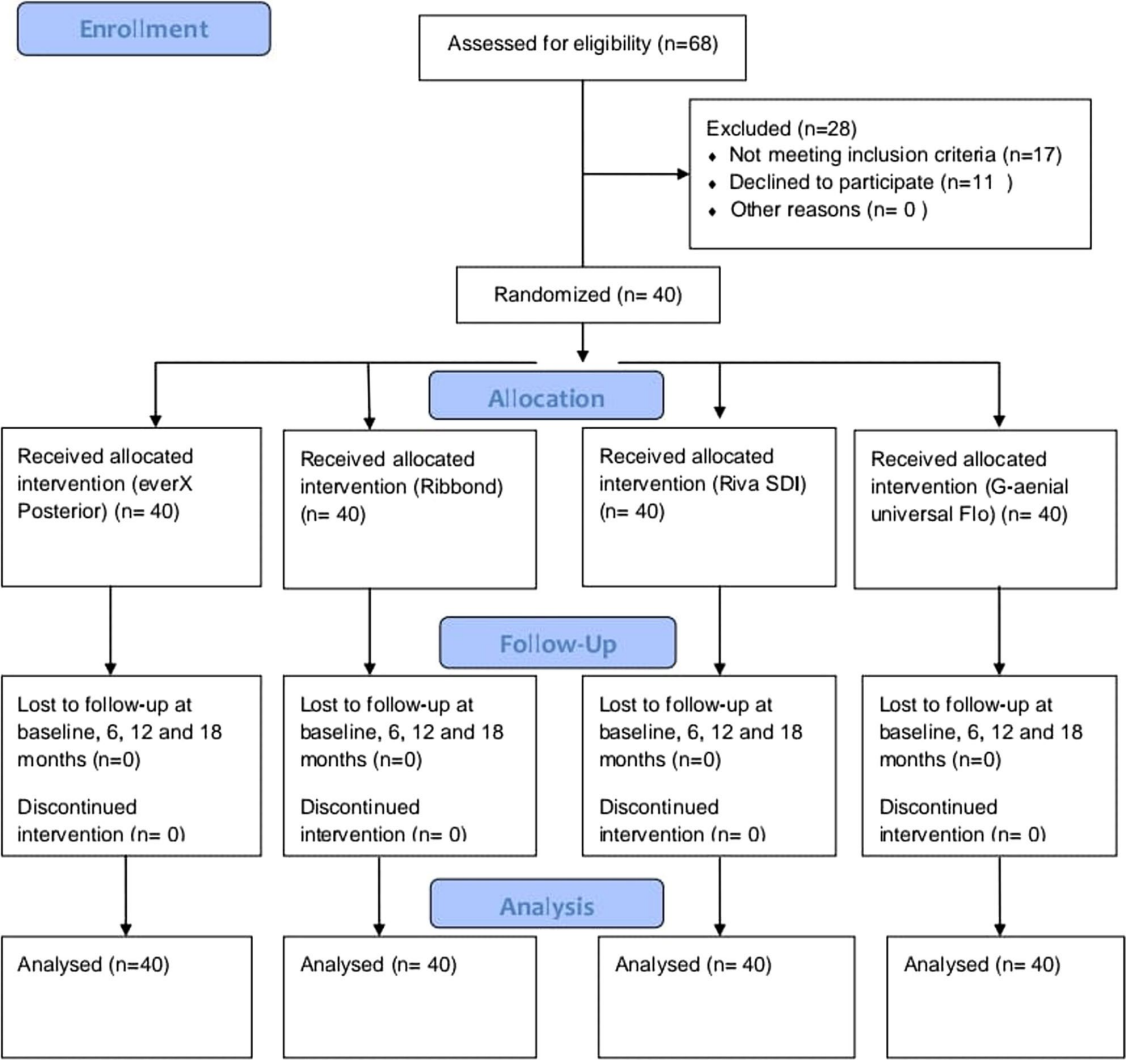
CONSORT flow chart

A randomized, comparative clinical trial using a split-mouth design was used to carry out this study. This study adheres to CONSORT guidelines. The 2025 CONSORT statement, which consolidated standards for reporting trials, served as the basis for the study’s design (Fig. 1) [14].

## Study setting

This study was carried out at the clinic of Department of Restorative Dentistry, Faculty of Dentistry, Tanta University.

## Sample size

**Fig. 2:**
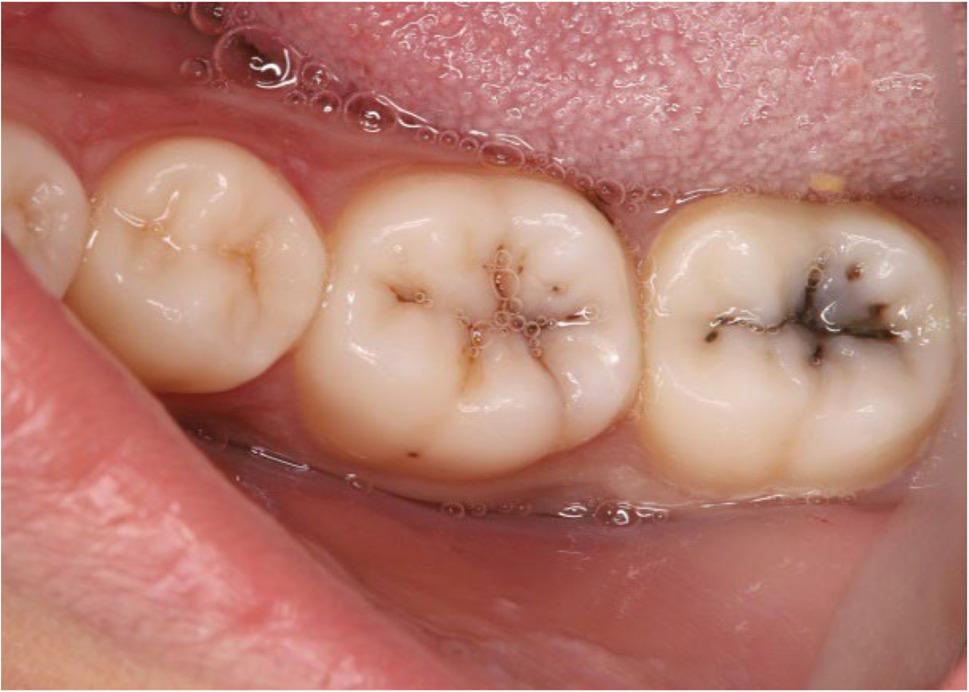
Preoperative intraoral photograph of occlusal class I carious lesions of lower right permanent first and second molars

The sample size was computed using the G power version 3.1.9.6 software statistical tool. The alpha value was set at 0.05, with a statistical power of 80% and an effect size of 0.307. The study minimally needed 20 posterior composite restorations in each group distributed on 20 patients who had at least four Class I cavities in vital posterior teeth (Fig. 2). The sample size was intentionally oversized, with a total of 160 restorations split among 40 patients. This was done to account for potential drop-outs and ensure adequate statistical power and internal validity.

## Ethical considerations

Selected patients were informed of all aspects of the treatment process, including the benefits, drawbacks, expected outcomes, and anticipated complications. Following the guidelines on human research adopted by the Research Ethics committee, Faculty of Dentistry, Tanta University, which approved the protocol of this clinical research after fulfilling all committee requirements with code #R-RD-03-25-3194. All participants were asked to provide their informed consent and assigned. The standards set out in the Declaration of Helsinki were adhered to in every aspect of this research.

## Patient selection

After a comprehensive set of inclusion and exclusion criteria, forty participants, ranging in age from 35 to 45, were chosen to participate in the current study. The participants were recruited from the Restorative Dentistry Department clinic at Tanta University’s Faculty of Dentistry. Patients were educated on proper dental hygiene practices prior to restorative procedures, and they were sent to the periodontics department for scaling and polishing as necessary.

### Inclusion criteria

- Maintaining quality dental care
- had at least four occlusal Class I carious lesions in vital posterior teeth
- Optimal periodontal health with no periodontal pockets > 3 mm or clinical attachment loss
- Time available for subsequent appointments

## Exclusion criteria

- Uncooperative patient
- Endodontically treated or non-vital teeth
- Para functional habits such as tooth clenching or grinding
- Pregnancy

## Randomization and allocation concealment

By utilizing (www.randomization.com), a randomization list has been generated. Each participant received four posterior composite restorations using the split-mouth approach; each patient was given an identification code and four possibilities for composite restorations were chosen at random from a list. Opaque sealed envelopes containing numbered cards were used to conceal the allocation. The envelope was made impenetrable to bright light by placing aluminum foil inside. A secretary unrelated to the restorative procedure gave each patient their own sealed opaque envelope. The envelopes contained a link that showed the restoration strategy used together with the randomization code that assigned each person to a group on the day of the operation [15].

### Blinding

It was a double-blind trial as both the patients and the evaluators who weren’t involved in the restoration processes were unaware which groups were being examined; however, the operator wasn’t blinded to either group.

### Restorative procedures

**Fig. 3:**
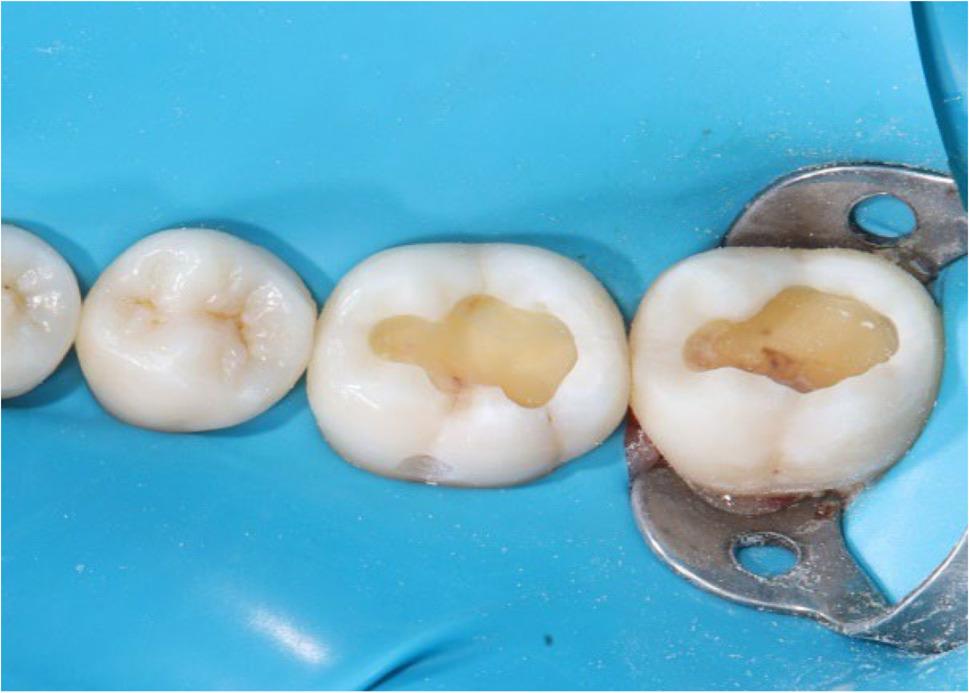
Intraoral photograph after removal of carious lesion and completing the cavity preparation

The principal investigator executed all steps involved in preparing the cavity and performing the restoration procedures. Prior to study initiation, the operator underwent a calibration phase involving standardized 10 pilot restorations to ensure consistent clinical technique across all tested materials. A rubber dam with a thickness of 0.010 inches was used to entirely isolate the restorative field. Occlusal class I cavities were made in accordance with caries extension, and their depth was at 4 to 5 millimeters measured by a calibrated periodontal probe intra-operatively from the floor of the cavity to the cavosurface margin. Bucco-lingual dimensions of Class I cavities were measured at baseline using a calibrated periodontal probe to ensure that the cavities were categorized as large and wide (bucco-lingual dimensions were about more than one half to two third the intercuspal distance). Tungsten carbide burs (Komet^®^ H33L, Lemgo, Germany) held in a high-speed contra-angled handpiece with a water-cooling system were used to prepare all of the cavities (Fig. 3). The prepared cavity margins were finished with finishing diamond stones that were color-coded in yellow to smooth the cavosurface margins without creating a defined bevel. Selective enamel etching was performed using 37% phosphoric acid gel (N-Etch, Ivoclar, Vivadent, Schaan, Liechtenstein) for twenty seconds according to the manufacturer’s recommendations; then the gel was rinsed off and the tooth was dried [16].

Following the manufacturer’s instructions, a disposable micro-brush was used to apply Tetric N-Bond universal adhesive (a one-step self-etch adhesive) to all surfaces of the cavities in each group. The material was then rubbed for 20 s before being gently dried with steam of air for five seconds, and then light curing was allowed for ten seconds using a light-emitting diode device (Woodpecker Dental LED D, wireless LED lamp curing light, China) with output intensity (850–1000 mw/cm²) in standard continuous mode. The curing tip was positioned perpendicular to the restoration surface. The tip-to-material distance was maintained at approximately 1 mm. The enamel etching and adhesive application protocol were performed identically for all four groups to ensure standardization of bonding procedures and eliminate adhesive-related variability. It was necessary to periodically measure the output intensity and durability of the LED curing light using a radiometer (Demetron Research Corp., Danburg, CT, USA) after each batch of ten composite restorations was cured [17].

Each patient received four different bi-layer biomimetic composite resins. A total of 160 posterior composite restorations were divided into four equal groups (*n* = 40) as follows: Group I: short fiber reinforced composite (everX Posterior, GC Corporation, Tokyo, Japan); Group II: long fiber reinforced composite (polyethylene fibers, Ribbond Inc., USA); Group III: resin-modified glass ionomer cement (RMGIC) (Riva SDI, Bayswater, VIC, Australia); and Group IV: flowable composite (G-aenial universal Flo, GC Corp., Tokyo, Japan).

### Group I

The short-fiber reinforced composite (everX posterior) was placed and light-cured in accordance with the manufacturer’s instructions for 20 s. The everX Posterior base filling was carefully applied so as not to extend beyond the outside cavo-surface boundaries of the prepared cavities to ensure that there is a 2 mm of occlusal space remaining for the application of the overlaying nanohybrid resin composite (Tetric Evo Ceram) [18].

### Group II

Two lengths of ribbond (polyethylene fibers, Ribbond Inc., Seattle, WA 9101, USA) were snipped with specialized shears known as ribbond scissors. In order to ensure that the polyethylene fibers were precisely cut to fit the cavity, a calibrated periodontal probe was used to assess the mesio-distal distance and bucco-lingual width of the cavities. After that, we used a patient napkin to wipe up any excess resin after wetting the fibers with an unfilled resin (Ribbond Wetting resin). The overlying nanohybrid composite (Tetric Evo Ceram) was applied directly over the fiber with no additional intermediate restorative material and was light-cured simultaneously according to manufacturer instructions. This simultaneous curing approach ensured integration of the fiber within the composite matrix while preventing formation of an excessively thick or poorly polymerized intermediate resin layer [19].

### Group III

Riva Conditioner (20% polyacrylic acid, SDI, Bayswater, VIC, Australia) was applied to the prepared cavities for 10 s, then rinsed thoroughly with water, and excess water was removed. The Riva capsules were activated by pushing the plunger until it was flush with the body. Resin-modified glass ionomer cement (RMGIC) (Riva SDI, Bayswater, VIC, Australia) that required mixing was manipulated in an amalgamator (Ultramat 2, SDI, Bayswater, VIC, Australia) (4000–4800 rpm) for 10 s. Once mixed, the material was injected into the prepared cavities using a Riva applicator. Then, it was light-cured for 20 s. Tetric Evo Ceram was applied to the leftover top part of the cavity and then let to light cure for 10 s [20].

### Group IV

A flowable composite (G-aenial universal Flo) was applied to the floor of prepared cavities with the dispensing tip and light-cured for 20 s, making sure to leave no more than 2 mm of occlusal space for application of the final cap layer of nanohybrid composite as other groups [21].

**Fig. 4:**
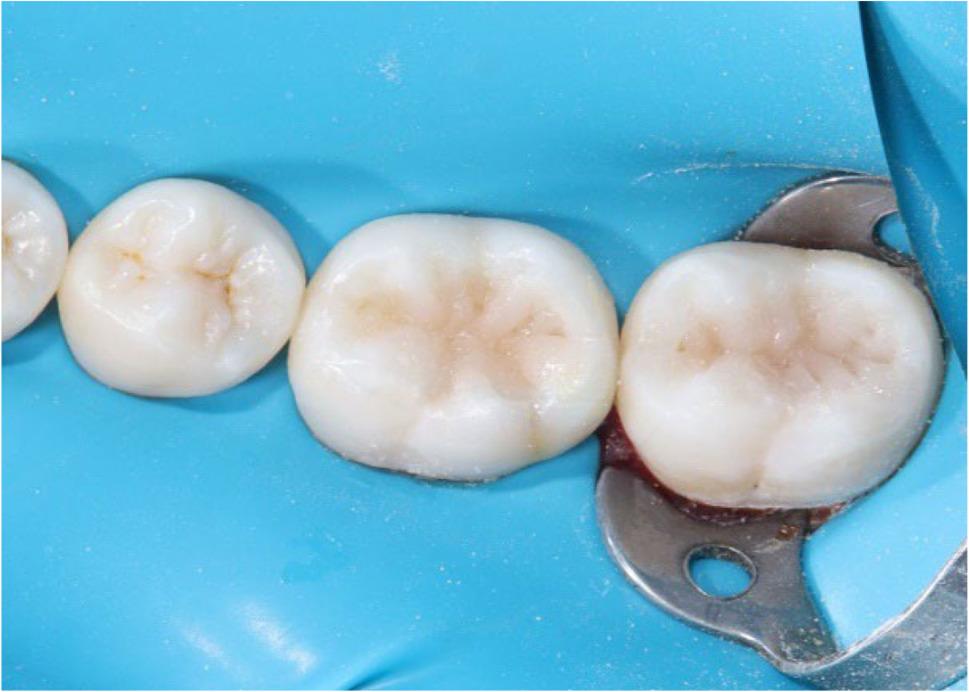
Intraoral photograph after composite placement of lower right permanent first and second molars

All of the restoration methods were standardized and intended to have an identical degree of curing by using the same light-curing unit throughout the trial. The curing tip was positioned perpendicular to the restoration surface, while the tip-to-material distance was maintained at approximately 1 mm. To conceal any evident flaws, such as over- or under-filling, the occlusal surface was sculpted to conform to the posterior teeth’s natural anatomy (Fig. 4) [22].

**Fig. 5:**
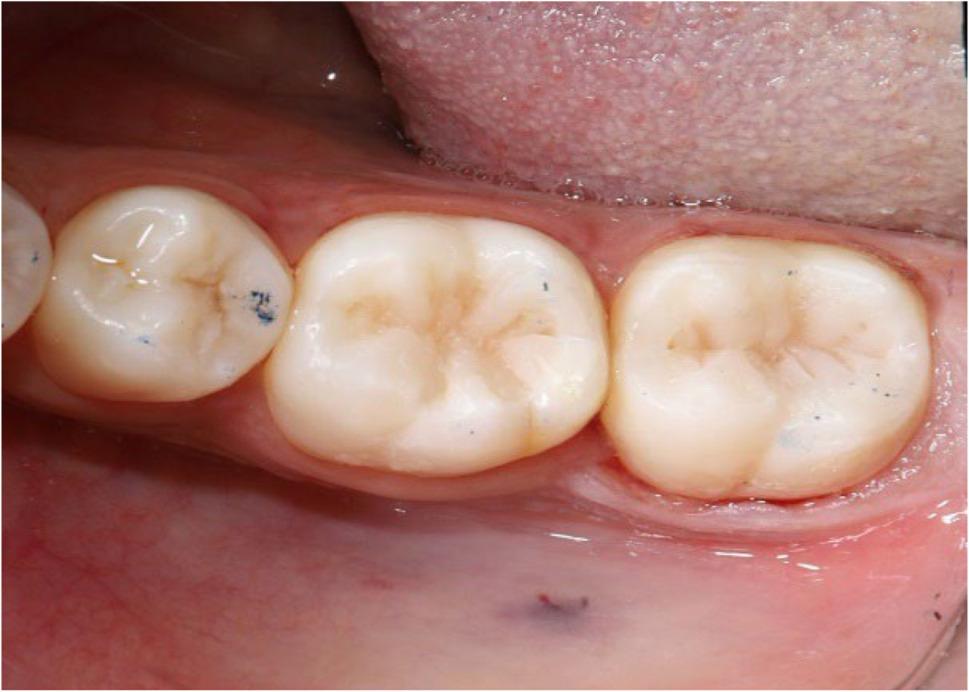
Intraoral photograph of final restorations of lower right first and second molar after finishing and polishing

After curing of composite resin restorations, finishing was performed immediately using water-cooled fine and super-fine diamond points (KG kit; Karebseb Ltd, Brazil). Articulating paper (Bausch, Nashua, NH, USA) was used to adjust occlusal contacts with the opposing teeth following the evaluation of centric and eccentric occlusion. Polishing was performed using polishing paste and aluminum oxide polishing cups and disks (Enhance finishing & polishing systems, Dentsply, Caulk, Milford) to achieve a highly polished surface (Fig. 5) [23].

### Evaluation procedures

All restorations were assessed clinically at baseline and at 6, 12, and 18 months according to the World Dental Federation (FDI) criteria, which are composed of five scores for each of the different criteria for aesthetic properties (surface and marginal staining), functional properties (fracture and retention, marginal adaptation), and biological properties (postoperative sensitivity, secondary caries) (Table 2). Evaluation sheets were used to record the patient’s scores at each follow-up period [24].

Two calibrated investigators who were not involved in the placement of the restorations evaluated the restorations clinically under a dental operating light, using an intraoral camera, flat-surfaced mouth mirrors, and a dental explorer. Prior to the evaluation, both examiners were calibrated to 100% agreement on 10 patients not included in this study. In the event of disagreement, a decision was reached by consensus. Restorations were scored using a scale of 1 to 5, where score 1 represented the ideal clinical situation, score 2 was clinically acceptable, score 3 was questionable but still clinically acceptable, and scores 4 and 5 were considered clinically unacceptable situations where the restoration has to be repaired or replaced [25].

As part of the assessment of postoperative hypersensitivity, patients were given a visual analogue scale (VAS) to measure the severity of their pain response and were also asked to verbally answer questions on the following: The evaluation also included questions about cold and/or heat sensitivity, as well as spontaneous pain that may or may not last. We also marked the line based on how much discomfort we felt while chewing or from other stimuli. An absolute value or percentage of the maximum pain imaginable, was displayed for pain intensity along a 100-mm line that ranged from 0 (no pain) to 100 (worst agony imaginable) [26].

**Table 2:** Descriptive FDI criteria for scoring restoration quality

Category	Parameters (sub Category)	Clinically Excellent (Score 1)	Clinically Good (Score 2)	Clinically Satisfactory ( Score 3)	clinically un satisfactory but reparable (Score 4)	Clinically Poor (Score 5) replacement necessary
Esthetic properties	Surface and marginal staining	No staining	Minor staining, easily removable	Moderate staining that may also present on other teeth, not esthetically unacceptable	Unacceptable pronounced staining; major intervention necessary for improvement	Severe staining; not accessible for intervention
Functional Properties	Fracture and retention	No fractures or cracks	Small hairline cracks	Two or more or larger hairline cracks and/or chipping (not affecting the marginal integrity or proximal contact)	Material chip fractures which damage marginal quality or approximal contacts; bulk fractures with partial loss	Partial or complete loss of restoration or multiple fracture
	Marginal adaptation	Harmonious outline, no gaps, no white or discolored lines	Small marginal fracture removable by polishing; slight ditching, slight step/flashes, minor irregularities	Several small marginal fractures; major irregularities, ditching, flash or step	Severe ditching or marginal fractures; larger irregularities or steps (repair necessary)	Restoration (complete or partial) is loose but in situ; generalized major gaps or irregularities
Biological Properties	Postoperative hypersensitivity	No hypersensitivity	Minor hypersensitivity for a limited period of time (< one week)	Moderate hypersensitivity lasting for less than six months ; mild sensitivity, no complains	Extreme hypersensitivity; cold sensitivity with typically accompanied by minor subjective symptoms	Acute pulpitis or non-vital tooth; endodontic treatment is mandatory
	Secondary caries	No secondary or primary caries	Small and localized demineralization	Larger areas of demineralization, dentin not exposed	Caries with cavitation and suspected undermined caries	Deep secondary caries or exposed dentin that is not accessible for repair

### Statistical Analysis

Data was gathered, tabulated, and statistically analyzed using the Statistical Package for the Social Sciences (SPSS) version 26 software program throughout the assessment periods. The distribution of criteria scores for the same group was compared at each assessment time using the Friedman test, while the distribution of scores between the four groups was compared at each clinical evaluation period using the Kruskal-Wallis test. Differences were deemed significant when the *p*-value was less than 0.05 and highly significant when the *p*-value was less than 0.001.

## Results

**Fig. 6:**
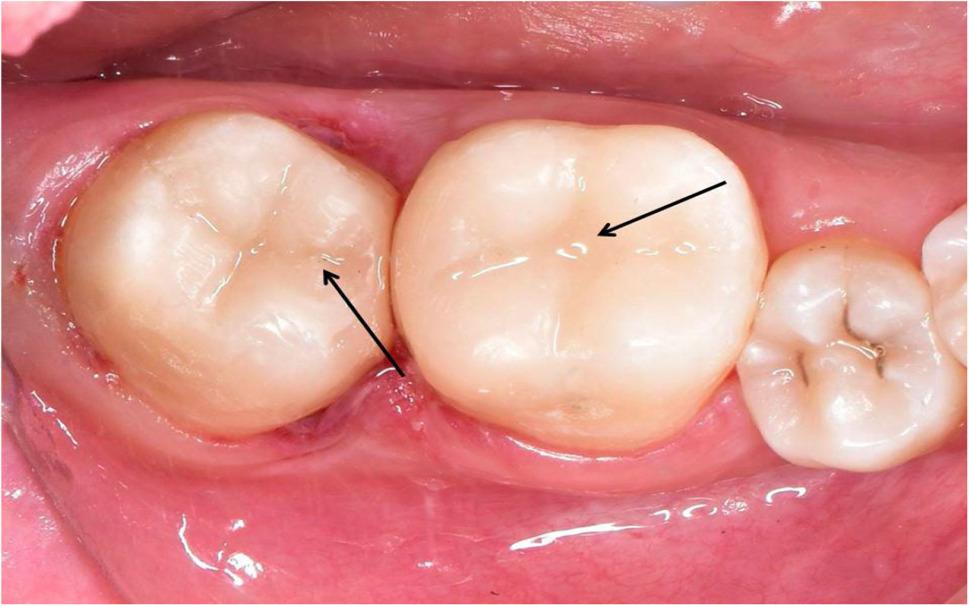
Clinical photo represents score 1 of surface and marginal staining for lower left first molar (Group I) and lower left second molar (Group II) at baseline

**Fig. 7:**
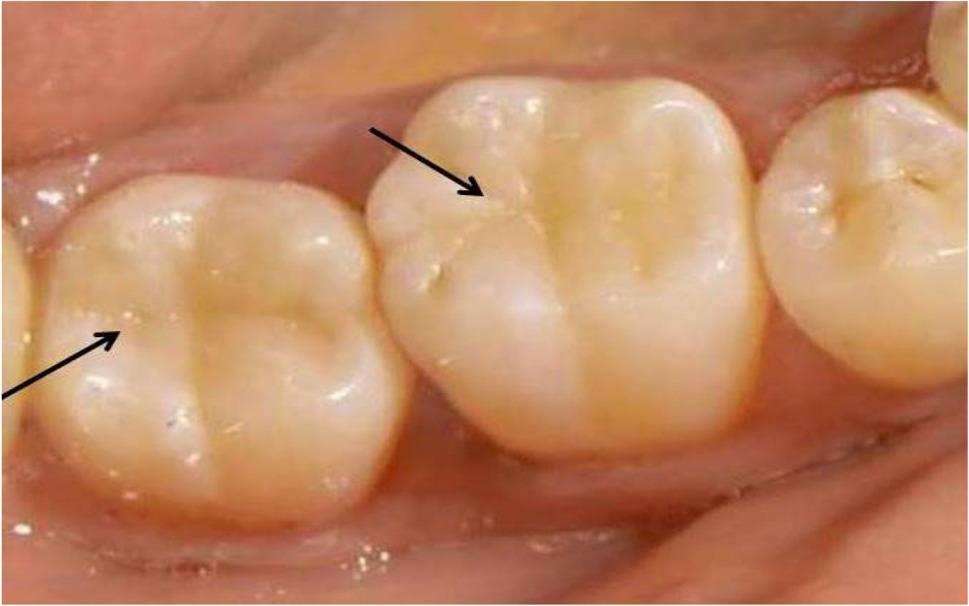
Clinical photo represents score 1 of surface and marginal staining for lower right first molar (Group III) and lower right second molar (Group IV) at 18 months follow up periods

**Fig. 8:**
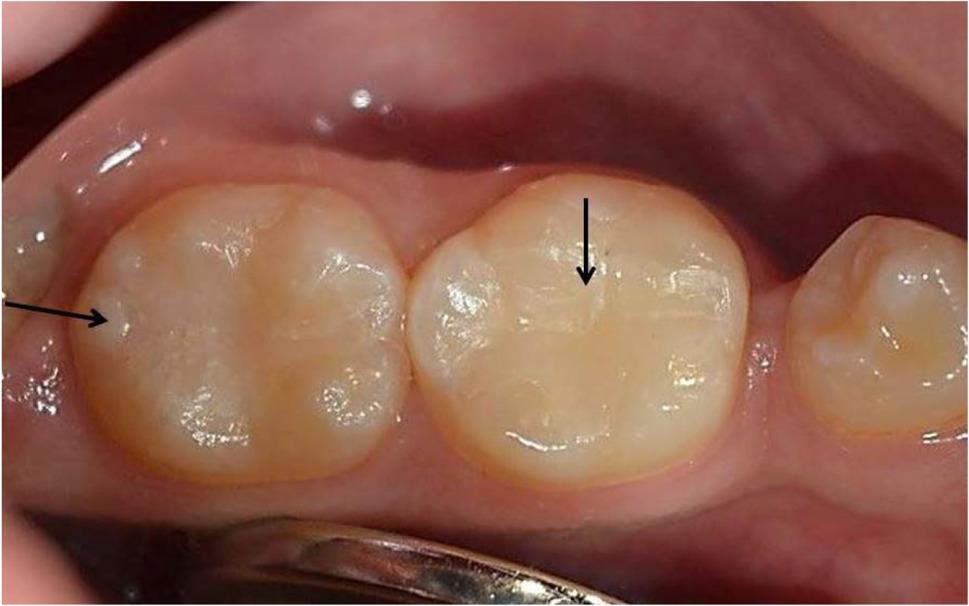
Clinical photo represents score 1 of marginal adaption for lower left first molar (Group II) and lower left second molar (Group III) at 18 months follow up periods

**Fig. 9:**
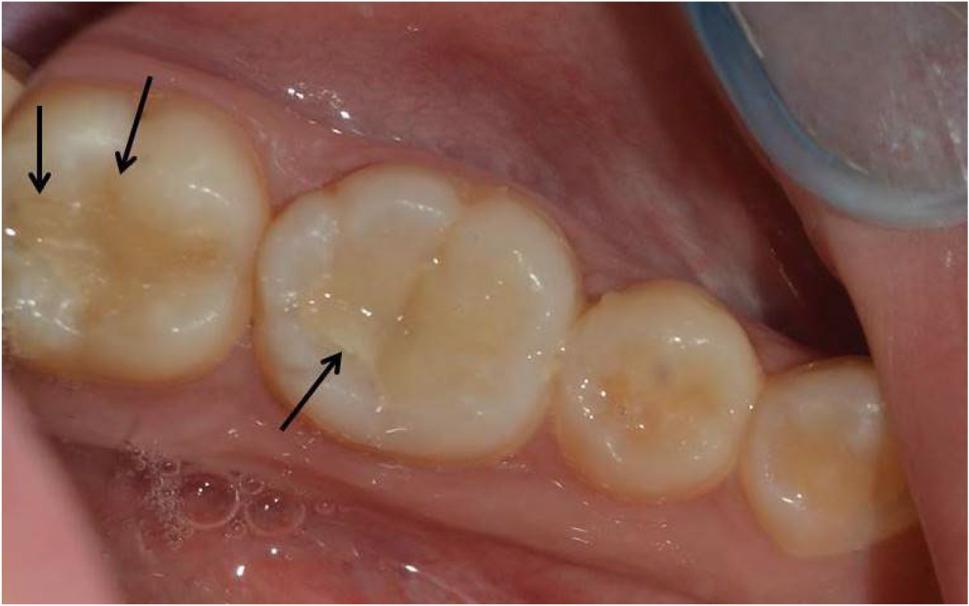
Clinical photo represents score 2 of marginal adaption for lower left first molar (Group I) and score 3 of marginal adaption for lower left second molar (Group IV) at 18 months follow up periods

**Fig. 10:**
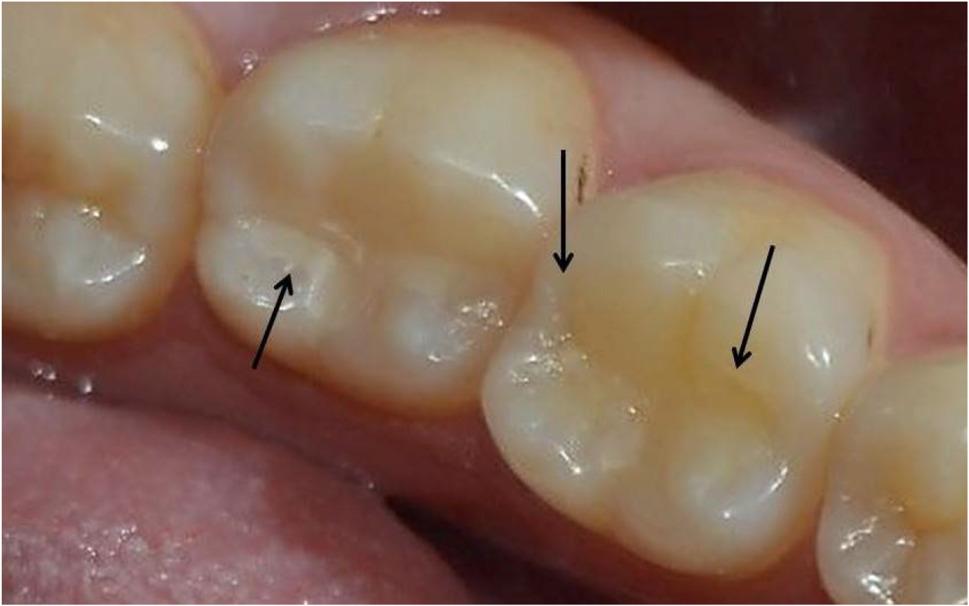
Clinical photo represents score 3 of marginal adaption for lower left first molar (Group VI) and score 2 of marginal adaption for lower left second molar (Group III) at 18 months follow up periods

The success rate for all groups was 100% by the end of the study after 18 months of follow-up periods. The scores of clinical assessments for the present study showed no statistically significant differences between the tested groups at baseline and after 6, 12, and 18 months (*p* > 0.05) by the Kruskal-Wallis test. Furthermore, across all groups and evaluation criteria, no significant changes were found over time (*p* > 0.05) by the Friedman test. Clinical intraoral photographs were displayed to elucidate the current results (Figs. 6, 7, 8, 9 and 10).

Table 3 presents the data of the clinical assessment for all criteria across the examined groups. The fracture and retention analyses reveal that the study’s whole follow-up period did not result in any restoration loss or fracture. All restorations had a score of 1 (fracture and retention rate of 100%).

**Table 3:** Data of clinical evaluation of all criteria in four tested groups (GI: everX, GII: Ribbond, GIII: Riva, GIV: G-aenial) at each follow-up period

Criterion	FDI Scores	baseline				After 6 months				After 12 months				After 18 months			
		GI	GII	GIII	GIV	GI	GII	GII	GIV	GI	GII	GIII	GIV	GI	GII	GIII	GIV
Fracture and retention	1 2 3 4 5	40 0 0 0 0	40 0 0 0 0	40 0 0 0 0	40 0 0 0 0	40 0 0 0 0	40 0 0 0 0	40 0 0 0 0	40 0 0 0 0	40 0 0 0 0	40 0 0 0 0	40 0 0 0 0	40 0 0 0 0	400 0 0 0	40 0 0 0 0	40 0 0 0 0	40 0 0 0 0
Surface and marginal staining	1 2 3 4 5	40 0 0 0 0	40 0 0 0 0	40 0 0 0 0	40 0 0 0 0	40 0 0 0 0	40 0 0 0 0	40 0 0 0 0	40 0 0 0 0	39 1 0 0 0	40 0 0 0 0	38 2 0 0 0	39 0 1 0 0	38 2 0 0 0	39 1 0 0 0	38 2 0 0 0	38 1 1 0 0
Marginal adaptation	1 2 3 4 5	40 0 0 0 0	40 0 0 0 0	40 0 0 0 0	40 0 0 0 0	40 0 0 0 0	40 0 0 0 0	40 0 0 0 0	40 0 0 0 0	38 2 0 0 0	39 1 0 0 0	38 2 0 0 0	38 1 1 0 0	37 2 1 0 0	38 1 1 0 0	38 2 0 0 0	37 2 1 0 0
Secondary caries	1 2 3 4 5	40 0 0 0 0	40 0 0 0 0	40 0 0 0 0	40 0 0 0 0	40 0 0 0 0	40 0 0 0 0	40 0 0 0 0	40 0 0 0 0	40 0 0 0 0	40 0 0 0 0	40 0 0 0 0	40 0 0 0 0	40 0 0 0 0	40 0 0 0 0	40 0 0 0 0	40 0 0 0 0
Postoperative hypersensitivity	1 2 3 4 5	31 9 0 0 0	34 6 0 0 0	36 4 0 0 0	32 8 0 0 0	40 0 0 0 0	40 0 0 0 0	40 0 0 0 0	40 0 0 0 0	40 0 0 0 0	40 0 0 0 0	40 0 0 0 0	40 0 0 0 0	40 0 0 0 0	40 0 0 0 0	40 0 0 0 0	40 0 0 0 0

Regarding the outcomes of the surface and marginal staining criteria, it was found after 12 months for group I that one restoration (2.5%) recorded a score of (2) compared to two restorations (5%) that recorded a score of (2) at 18 months. For group II, just one restoration (2.5%) had a score of (2) at 18 months. Two restorations (5%) of group III recorded a score of 2 at 12 and 18 months. For group IV, one restoration (2.5%) recorded a score of (3) at 12 months, while two restorations (5%) had scores of (2) and (3), respectively, at 18 months.

The Friedman test revealed no statistically significant differences in surface and marginal staining scores over the 18-month follow-up for each group I, II, III, and IV (p-values = 0.241, 0.801, 0.261, and 0.241 in that sequence). Furthermore, the Kruskal–Wallis test revealed that at 12 months and 18 months, respectively, the p-values were 0.355 and 0.438, indicating that there were no statistically significant differences between the four tested groups.

In regards to marginal adaption findings; for group I two restorations (5%) had score (2) after 12 months, while two restorations (5%) had score (2) and one restoration (2.5%) had score (3) at 18 months. For group II one restoration (2.5%) recorded score (2) at 12 months and two restorations (5%) recorded score (2) and (3) respectively. On the other hand, for group III; two restorations (5%) recorded score (2) after 12 and 18 months. In group IV; two restorations (5%) recorded score (2) and (3) respectively at 12 months and two restorations (5%) recorded score (2) and one restoration (2.5%) recorded score (3) after 12 months.

Intragroup comparisons using the Friedman test indicated that no statistically significant difference for each group I, II, III, and IV (p-values = 0.066, 0.11, 0.21, and 0.146 in that order). Intergroup comparisons at each follow-up interval using the Kruskal–Wallis test demonstrated no significant differences among the four tested restorative materials at baseline, 6 months, 12 months, or 18 months (baseline: *p* = 1.00, 6 months: *p* = 1.00, 12 months: *p* = 0.72, 18 months: *p* = 0.70).

Based on the data presented in Table 3, it can be concluded that no restorations experienced secondary caries during any of the assessment periods. All four groups maintained a perfect recording score (1) rating throughout the follow-up periods, hence no statistical analysis was carried out.

The results of the clinical evaluation of the hypersensitivity of the restored teeth were translated to scores as displayed in Table 3. All the collected data belong to the criteria of tested groups were evaluated at all follow-up periods as score (1) except at the baseline period following restoration which nine restorations (22.5%) of group I, six restorations (15%) of group II, four restorations (10%) of group III and eight restorations (20%) of group IV suffered from post-operative hypersensitivity and recorded score (2). No statistically significant difference was reported in hypersensitivity among the four groups at baseline ( *p* = 0.452).

## Discussion

Direct composite restorations have achieved widespread use to restore both anterior and posterior teeth as they are minimally invasive restorative procedures and therefore can preserve as much tooth tissue as possible while providing long-term clinical durability. The field of restorative dentistry greatly relies on the biomimetic approach. Numerous restorative materials having biomechanical properties comparable to those of the healthy tooth structure are required to replace lost tooth structures [10].

Various flowable resin, glass ionomer cement and fiber-reinforced composite materials have gained prominence in being predominantly used as dentin replacement materials that can replace tooth tissue in a biomimetic manner. Nevertheless, traditional particle filler composites with improved wear resistance must be applied as a final cap to these materials. In this case, a bi-layered restoration is formed by using two materials, each of which has its own unique set of characteristics. As a result, a dynamic heterogeneous structure resembling a natural tooth tissue; enamel and dentin were created [27].

One of the improvements in the biomimetic dentistry is the fiber reinforcing of resin composite, which involves adding fibers to the filler system either internally or externally to prevent cracks from spreading and relief the polymerization stresses. Reinforcing resin composites with short, discontinuous glass fibers serve as an interior reinforcement (Ever X Posterior), while polyethylene fibers serve as an exterior reinforcement (Ribbond-Ultra fibers) [28].

The components of ever X posterior include a resin matrix, inorganic fillers, and short e-glass fibers distributed in a random pattern within the matrix similar to dentin, which is composed of collagen fibers embedded in a matrix, the fibers serve as a reinforcing medium. In addition, fiber reinforced resin composite (Ever X Posterior) can improve mechanical performance by absorbing stresses and dispersing energy similarly to dentin [29].

Improved toughness and durability in high stress bearing regions have resulted from the implementation of ribbon fiber reinforcing systems in resin composites. To optimize the clinical efficiency, the polyethylene fibers were initially wetted in a plain unfilled resin liquid, as suggested by the manufacturer to increase its wettability, and impregnation within the resin composite. The pulpal floor was covered with horizontally laid polyethylene fibers, which increased the load bearing capacity, distributed forces evenly over the enormous surface area, and relieved stresses caused by polymerization shrinkage [10].

For this research, the manufacturer suggests adding a nanohybrid composite surface layer over a fiber-reinforced composite for better restoration results. Restorations were be more functionally robust and aesthetically pleasing, increasing the clinical success rates, by integrating the surface qualities of the nanohybrid composite with the strength and support of the fiber-reinforced composite. Because of its two-layer design, this restoration is a biomimetic model of a natural tooth [30].

The present investigation utilized the Class I cavity design due to its clinical resemblance to challenging cavity preparation and restoration. Microleakage, which frequently causes postoperative sensitivity, is more likely to occur due to the high configuration factor of these cavities, which hinders the flow of composite resin during polymerization shrinkage and increases contraction stresses over the bonding interface [31].

Due to its extensive use for evaluation of composite restorations, the FDI criteria were utilized for the clinical assessment of the restorations’ performance. Therefore, in order to make this randomized clinical study more credible, it was decided to incorporate it and pool its results with any future systematic reviews on fiber reinforced composite resins. Although the overall clinical results were satisfactory, the FDI evaluation criteria are more sensitive to modest differences in the clinical outcomes [32].

Patient factors such as caries risk, occlusal load, oral hygiene, and para-functional habits may impact restoration survival. Certain inclusion and exclusion criteria were used to choose the present participants, which may have minimized confounding influences and contributed to the uniformly high survival rates in the present study. Good dental hygiene with low caries risk and the absence of periodontal disease were observed in the participants. It is recommended that these individuals have a minimum of four posterior teeth with occlusal carious lesions. So, all restorations were subjected to the same environmental conditions. To eliminate the risks associated with younger individuals, such as high pulp horns, large pulp chambers, and hidden microscopic pulpal exposures, the chosen patients were between the ages of 35 and 45 [33, 34].

A randomized split mouth clinical trial was performed in the present study to remove a lot of inter-individual variability from the estimates of the treatment effect such as oral hygiene, diet and brushing habits. Randomization and allocation concealment is critical to the design of randomized clinical trials to avoid selection bias. The present study was double-blinded in order to eliminate investigator or patient-related bias. The restorations in this study were carried out under similar conditions by one operator, which greatly guarantees the accuracy of the results and increases the authenticity of present study [35].

Clinical assessment of different bi-layer biomimetic strategies of composite resin in direct posterior restorations revealed good outcomes, with predominantly clinically acceptable scores after 18 months. The success of the posterior restorations in several clinical trials is indicated by its longevity and durability in the oral cavity. Consequently, the most crucial evaluation criterion is retention and restoration fracture. In this investigation, after 18 months of clinical interventions, it had been found that 100% of the restorations were retained without fracture. This is a fairly objective criterion for estimating the clinical efficacy of the applied adhesive and restorative materials. Since this criterion is not dependent on the subjective evaluation of the examiner, this is the gold standard for diagnostics and the clearest indicator of a restoration that has failed [36].

There were no notable variations in retention and fracture scores among the four groups that were examined in the study. This could be attributed to the adequate mechanical properties of fiber-reinforced resin composites, glass inomer cement, flowable and nanohybrid resin composite (Tetric Evo Ceram), as well as the exclusion of participants with para-functional habits. This confirms what earlier studies have found: para-functional behaviors greatly impact the likelihood of posterior restorations surviving, especially when it comes to restoration fracture. In addition, the results may be explained by the harmonic functioning of the tooth substrate and restorative composite under load, which is achieved through the effective transmission of stresses between various bilayer biomimetic materials and the applied surface layer of nanohybrid composite. Also, the standardized Class I cavity geometry inherently provides substantial mechanical retention, which may have contributed to the 100% restoration survival rate observed across all groups, independent of the type of base layer material used [37, 38].

The results are in accordance with Suhasini K et al., [39] who performed a randomized clinical trial to evaluate the efficacy of Class I nanohybrid composite restorations using flow-able composite and resin modified glass-ionomer as dentin substitute. The results confirmed that the restorations still performed well clinically after twelve months follow up, and the researchers concluded that the glass-ionomer and flowable composite were crucial in reducing stresses caused by polymerization shrinkage through the elastic bonding concept, which in turn increased the restorations’ longevity and favorable outcomes.

On contrary, Nguyen AD et al., [40] evaluated Class I and Class II restorations using a flowable composite as an intermediate layer through a narrative assessment of clinical data. They reported that the restorations with and without an intermediate layer of flowable composite did not differ significantly in terms of annual failure rates, and the use of flowable composites did not appear to impact the clinical lifespan of direct composite restorations.

Also, Van de Sande FH et al., [41] assessed the 18-year survival of posterior composite restorations with and without a base of glass ionomer cement and revealed that the presence of a base did not impact the survival rates of the restorations, so it could be kept without clinical issues. However, it should be noted that this measure does not improve survival rates. Ultimately, they concluded that patient risk profiles and operator characteristics affect restoration survival in addition to tooth and cavity variables.

On the other hand, these results of fiber-reinforced composites were consistent with those of Mohamed MH et al., [42] who evaluated the clinical performance of short, long fiber-reinforced, and conventional resin composites over 18 months and reported that all tested restorations had the same excellent scores without fracture by the end of the trial period. Also, Metwaly AA et al., [43] concluded that 100% of fiber-reinforced and bulk-fill resin composite restorations demonstrated satisfactory clinical outcomes and were retained with no fracture score during a 24-month follow-up period.

One possible explanation for the observed outcomes is the beneficial strengthening effect in various directions brought about by the efficient transfer of stress throughout the resin matrix after curing and in between the fibers. Individual fibers serve as a barrier for cracks because of the fibers’ arrangement in a closely woven framework with nodal connections. In addition, the polyethylene fibers were arranged against the cavity walls in this investigation to simulate the dentino-enamel complex. The tooth’s substructure and restorative composite can then support each other when subjected to stresses [44].

Bijelic-Donova J et al., [10] performed a meta-analysis and systematic review to determine whether direct composite restorations reinforced with short glass fiber (Ever X) or polyethylene fiber (Ribbond) substructure have different fracture resistance and fracture mode and compared the two types of fiber reinforcement. They concluded that both kinds of fibers strengthen teeth against cracks, and they work just as well when applied to teeth with weak structural support, and they recommended that a single layer of Ribbond might be used for the cavity floor, while EverX Posterior would be better suited to restore lost dentin in an anatomically formed manner.

However, Negm HM et al., [45] and Youssef HH et al., [46] assessed the fracture resistance of fiber-reinforced resin composite restorations in permanent molars and found that, when compared to flowable composite (Tetric N-flow) and ultra-polyethylene fiber tape (Ribbond-Ultra), fiber-reinforced composite teeth (EverX Posterior) exhibited the greatest fracture resistance durability. On the other hand, they recommended that the polyethylene fiber tapes aren’t as efficient and need more effort to apply. The observed inconsistency may be ascribed to heterogeneity in experimental methodologies, including variations in study design, the type of resin composite employed in conjunction with polyethylene fiber reinforcement (Ribbond^®^), differences in adhesive systems and tooth substrate characteristics that could have influenced the reported outcomes.

As regards the current clinical assessments of marginal adaptation and staining of all tested restorations, which are often related to each other and pose a significant issue in restorative material evaluation. The results demonstrated that the investigated materials had low polymerization shrinkage and shrinkage stresses; hence, the scores were not statistically different across groups. Due to their reduced modulus of elasticity, the investigated materials can operate as elastic buffers or stress-breaking barriers to reduce polymerization shrinkage stresses and improve marginal adaptability [47].

Results from the marginal adaptation of SFRC were in line with those from earlier research. The stress-relaxation mechanism is thought to be affected by the quantity, aspect ratio, and fiber orientation. In terms of fibers, the aspect ratio is the ratio of fiber length to diameter. The fibers will entwine once the cavity is filled with EverX Posterior due to their high aspect ratio. Along the fiber-matrix interface, the polymerization shrinking also decreased. Thus, the organic matrix interspersed with the fibers can shrink while maintaining its initial horizontal dimensions [48].

The translucency of Ever X Posterior also lets light curing in at a greater depth, which speeds up the polymerization process and creates the best possible marginal seal. This is in agreement with the findings of Salem et al., [49] who also found no significant difference in terms of marginal adaption after 12 months clinical evaluation of bi-layered structure using a fiber reinforced composite and a nanohybrid capping layer. Their research indicated that the intertwined short glass fibers absorbed the stresses acting upon the tooth-restoration interface, which was the site of the majority of the polymerization stresses.

This study’s findings on polyethylene fibers were in line with those of Özüdoğru and Tosun [50]., who had previously postulated that the use of polyethylene fibers to an elastic shock absorber formed at the base of the cavity. This shock absorber aids in reducing stresses during polymerization and improving the material’s marginal integrity. In comparison to restorations devoid of fibers, those containing fibers can reduce microleakage around the restoration. This is because fibers have a low flexural modulus and a high elastic modulus, both of which help to alleviate stresses that build up at the dentin/resin interface. However, Hasija MK [51]., evaluated the effect on marginal adaptation of class II composite restorations reinforced with polyethylene fibers and concluded that the addition of polyethylene fiber in composite material does not improve the marginal adaptation. The discrepancy of these findings may be explained by methodological differences, as this study was conducted under controlled laboratory conditions with different cavity configurations that simulate isolated stress factors rather than the complex intraoral environment and do not account for factors such as dentin hydration, pulpal pressure, masticatory adaptation, and biofilm dynamics.

The outcomes regarding marginal adaptation and discoloration of resin modified GIC and flowable resin groups are in consistent with Suhasini K et al., [39] Also, Nguyen KV et al., [52] conducted a systematic review on clinical performance of resin composite restorations laminated with GIC and flowable resin and reported that there was no statistically significant difference between the two groups and both restorations are clinically acceptable. By contrast, Gernhardt CR et al., [53] and Miranda SB et al., [54] conducted numerous clinical trials, including long-term meta-analyses, and reported that there was no statistically significant clinical advantage for additional intermediate layers in terms of marginal adaptation, postoperative sensitivity, or restoration survival when compared to conventional composite restorations.

The excellent outcomes of the secondary caries were ascribed to reasons, such as the relatively short time frame of the monitoring period. Maintaining good dental and oral hygiene was also strongly emphasized by the participants. In addition to advancements in adhesive tactics and restoration materials, the accuracy of proper manipulation of restorative materials is extremely important. Irrespective of the condition of marginal integrity, patient-related factors are the primary determinants of secondary caries formation [42].

Nedeljkovic I et al., [55] included individuals with a high caries index and poor oral hygiene in their study, and they verified that patients at high risk of caries were associated with restoration failure caused by secondary caries. Additionally, all of the materials in the current study effectively prevented secondary caries, as evidenced by their reported ratings of excellent marginal adaption.

These results are consistent with earlier clinical investigations that have also revealed no evidence of secondary caries. Tekçe N et al., [56] reported that no secondary caries was observed in direct posterior composite restorations with and without short glass-fiber-reinforced over 3-year results. Manjunath V et al., [57] conducted 12 months randomized clinical trial and revealed that both fiber-reinforced composite and conventional composite restorations had no secondary caries.

Contrary to our findings, Tanner J et al., [58] found that one fiber reinforced composite restoration failed due to secondary caries in their study, while Bijelic-Donova J et al., [59] reported that two fiber reinforced composite restorations needed to be repaired or replaced for the same cause. This could have been influenced by patient-related factors, such as inadequate oral hygiene during the evaluation period.

Regarding the assessment of postoperative hypersensitivity, a subjective criterion since it relies on the patient’s complaint and is thought to be an issue with resin composite restorations. With the exception of the baseline evaluation period, no group in the current investigation exhibited postoperative hypersensitivity. There is evidence in the literature that the depth of the clinical cavity is the most important factor determining the likelihood of postoperative sensitivity, with sensitivity levels rising as cavity depth increases. All of the cavity depths in this investigation were modest, which explains the present results [60].

There is a dearth of published clinical trials utilizing various bilayer biomimetic techniques of composite resin at the present time; as far as we are aware, this study is one of the few. The early outcomes detectable within 18 months were marginal adaptation changes, surface staining, and early postoperative sensitivity, while long-term outcomes required extended follow-up to evaluate restoration survival, secondary caries, and retention and fracture. The null hypothesis of this study was accepted since no discernible variations were found in the clinical performance of the different tested bi-layer biomimetic composite resins.

### Limitations

Although the study time was considerably short, at 18 months it is acceptable and on pace with other clinical trials to provide insight into the behavior of restorative materials. Due to the different restoration processes used, operator blinding could not be implemented in this investigation, which could have produced bias. Although the design aimed to compare different bi-layer biomimetic strategies under standardized conditions, inclusion of a conventional single-layer composite-only group is recommended, as it could have provided additional comparative insight into the incremental benefit of intermediate stress-modulating layers. Future randomized clinical trials should assess a larger number of posterior restorations over an extended duration in more standardized complex cavities confirmed by radiographic examination, considering intra-patient variability to ascertain the long-term performance.

## Conclusions

It is possible to derive the following findings within the limitations of this 18-month randomized clinical study:

- All tested bilayer biomimetic composite resins have comparable excellent short-term clinical performance by the end of the study period.
- Reinforcing resin composites with either short glass or polyethylene fibers might be an effective method for restoring posterior cavities.

### Clinical consideration

Although all bi-layer biomimetic strategies demonstrated comparable short-term clinical outcomes, material-specific properties should guide clinical decision-making. RMGIC offers benefits such as fluoride release, chemical adhesion to dentin, relative moisture tolerance, and simplified handling, which may be particularly beneficial in patients with moderate to high caries risk. Fiber-reinforced composites provide enhanced fracture resistance potential and stress distribution; however, they are more technique-sensitive, require additional chairside time, and may increase procedural cost. Flowable composite presents ease of placement, improved cavity adaptation, and time efficiency, making them suitable for routine clinical applications. Consequently, clinicians should balance mechanical rationale with handling characteristics, operator experience, patient caries risk profile, and cost-effectiveness when selecting the most appropriate biomimetic base material.

## Data Availability

The datasets generated and/or analyzed during the current study are not publicly available due to patients’ privacy but are available from the corresponding author upon reasonable request.
